# Supplementary material for: Genetic comparisons of interleukin-17 reveal a framework for complex signaling evolution
Source: bioRxiv. 2026 Apr 14:2026.04.13.718218. Preprint. [Version 1] doi: 10.64898/2026.04.13.718218 (PMC13105070; doi:10.64898/2026.04.13.718218)
Supplement: Supplement 3 [file media-3.zip › Supplemental files for paper/ERC/IL17xIL17_comparisons/IL17RB_IL17RA.pdf]

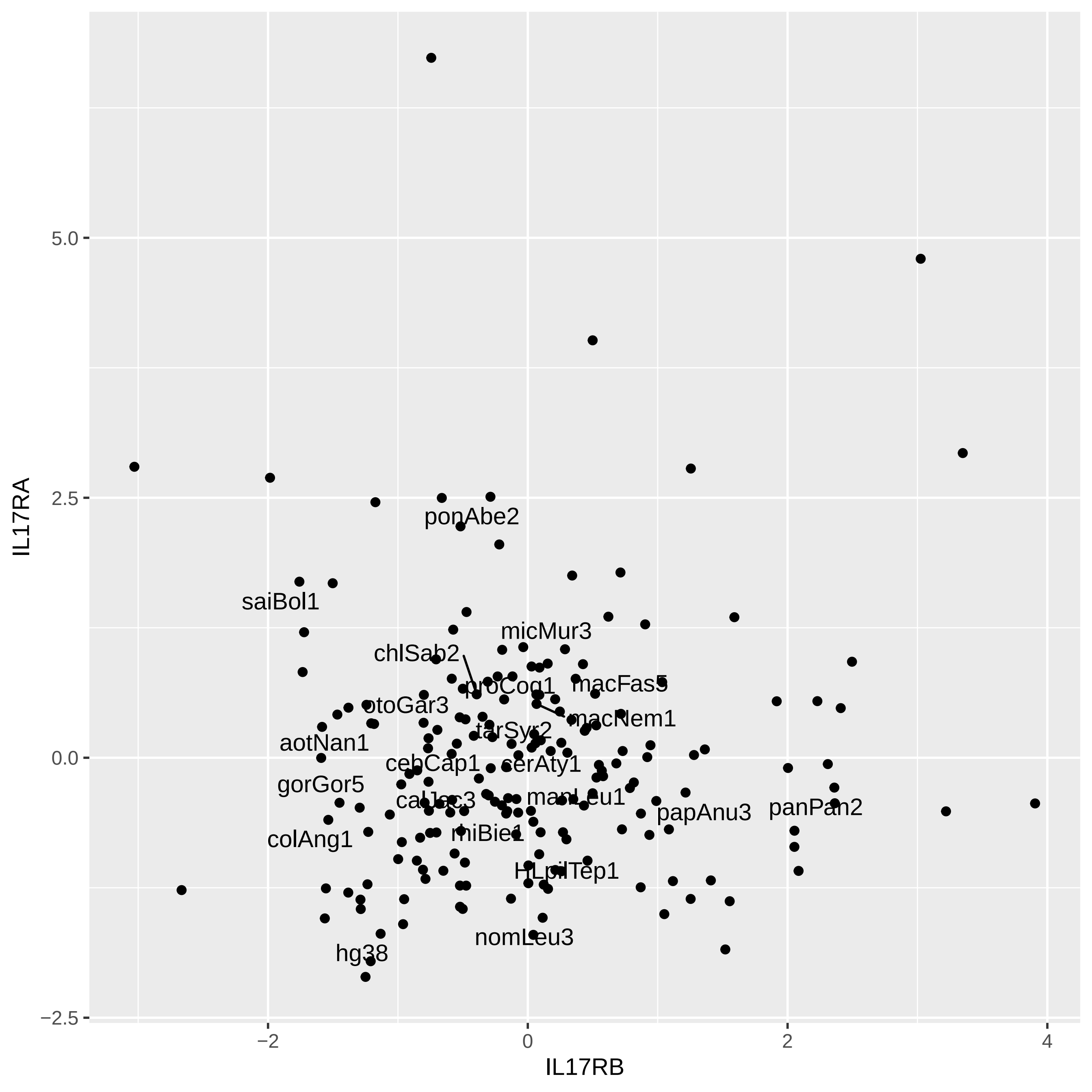

IL17RA
IL17RB
5.0
2.5
0.0
−2.5
−2
0
2
4
ponAbe2
saiBol1
micMur3
chlSab2
proCog1
macFas5
otoGar3
macNem1
tarSyr2
aotNan1
cebCap1
cerAty1
gorGor5
calJac3
manLeu1
papAnu3
panPan2
colAng1
miBie1
HLpilTep1
nomLeu3
hg38
